# Evaluation of Early Warning Scores on In-Hospital Mortality in COVID-19 Patients: A Tertiary Hospital Study from Taiwan

**DOI:** 10.3390/medicina59030464

**Published:** 2023-02-26

**Authors:** Weide Tsai, Chun Chen, Szu-Yang Jo, Chien-Han Hsiao, Ding-Kuo Chien, Wen-Han Chang, Tse-Hao Chen

**Affiliations:** 1 Department of Emergency Medicine, MacKay Memorial Hospital, Taipei 104, Taiwan; 2 Department of Medicine, MacKay Medical College, New Taipei City 252, Taiwan; 3 MacKay Junior College of Medicine, Nursing, and Management, Taipei 112, Taiwan; 4 Department of Linguistics, Indiana University, Bloomington, IN 47405, USA; 5 Institute of Mechatronic Engineering, National Taipei University of Technology, Taipei 106, Taiwan; 6 Graduate Institute of Injury Prevention and Control, College of Public Health and Nutrition, Taipei Medical University, Taipei 110, Taiwan

**Keywords:** COVID-19, mortality, early warning scores, rapid emergency medicine score, REMS

## Abstract

Coronavirus disease 2019 (COVID-19) remains a global pandemic. Early warning scores (EWS) are used to identify potential clinical deterioration, and this study evaluated the ability of the Rapid Emergency Medicine score (REMS), National Early Warning Score (NEWS), and Modified EWS (MEWS) to predict in-hospital mortality in COVID-19 patients. This study retrospectively analyzed data from COVID-19 patients who presented to the emergency department and were hospitalized between 1 May and 31 July 2021. The area under curve (AUC) was calculated to compare predictive performance of the three EWS. Data from 306 COVID-19 patients (61 ± 15 years, 53% male) were included for analysis. REMS had the highest AUC for in-hospital mortality (AUC: 0.773, 95% CI: 0.69–0.85), followed by NEWS (AUC: 0.730, 95% CI: 0.64–0.82) and MEWS (AUC: 0.695, 95% CI: 0.60–0.79). The optimal cut-off value for REMS was 6.5 (sensitivity: 71.4%; specificity: 76.3%), with positive and negative predictive values of 27.9% and 95.4%, respectively. Computing REMS for COVID-19 patients who present to the emergency department can help identify those at risk of in-hospital mortality and facilitate early intervention, which can lead to better patient outcomes.

## 1. Introduction

The World Health Organization proclaimed the coronavirus disease 2019 (COVID-19) to be a global pandemic in March 2020 [1]; as of June 2022, about 548 million have been infected with COVID-19 and over 6.5 million have died. Currently, Taiwan is viewed as one of the countries that has successfully contained the notorious disease; however, a brutal outbreak occurred in May 2021 during which approximately 16,000 people had confirmed COVID-19 infection and 843 patients died. Hospital emergency departments (ED) were overloaded with COVID-19 patients during the outbreak, and early warning scores (EWS) were used to identify high-risk COVID-19 patients for immediate intervention before they could potentially deteriorate.

EWS were developed in the late 1990s and are based on the concept that a worsening clinical condition can be predicted by changes in basic physiological parameters. [2] The Modified EWS (MEWS) was first introduced in the United Kingdom and was widely accepted in the United States of America [3]. Subsequently, the MEWS was revised to the National Early Warning Score (NEWS) by the Royal College of Physicians in 2012 [4]. The Rapid Emergency Medicine score (REMS) was devised from the Rapid Acute Physiology score (RAPS) and introduced in 2004 [5]. REMS has been demonstrated to be superior to RAPS, and while other EWS have also been used, the aforementioned three are the most studied.

The MacKay Memorial Hospital is a tertiary medical center in the populous Taipei City, and proper infection control policies have been established since the COVID-19 pandemic began. The screening unit in the ED was capable of separating patients with COVID-19 and immediately operating COVID-19 polymerase chain reaction specimens. Quarantine wards were set up on an isolated floor in the hospital for patients with COVID-19 [6,7,8,9,10]. Moreover, the hospital provided medical care for critical patients in a specified COVID-19 intensive care unit. During the COVID-19 outbreak in Taiwan, this hospital was converted into a designated hospital for treating COVID-19 patients.

Hence, this study aimed to evaluate the discriminatory ability of MEWS, NEWS, and REMS in predicting in-hospital mortality among COVID-19 patients admitted to the MacKay Memorial Hospital.

## 2. Materials and Methods

### 2.1. Study Design

This retrospective study was conducted in the ED of a designated COVID-19 tertiary hospital in Taiwan and conforms to Transparent Reporting of a multivariable prediction model for Individual Prognosis or Diagnosis (TRIPOD) guidelines [11].

### 2.2. Study Population

The study population included adult (≥20 years old) COVID-19 patients who were admitted to the ED and hospitalized between May 1 and July 31 2021. COVID-19 infection was confirmed by polymerase chain reaction (PCR) analysis of nasopharyngeal specimens. Exclusion criteria included patients who (1) were previously de-isolated from COVID-19 infection; (2) were transferred from other hospital; (3) had COVID-19 infection but experienced an out-of-hospital cardiac arrest (OHCA); (4) were missing data on vital signs at initial triage; or (5) were missing demographic data.

### 2.3. Variables

This study retrieved data on variables, including age, sex, past medical history, and laboratory tests within 24 h of admission from an electronic medical record database using the International Classification of Diseases, Clinical Modification, 10th Edition (COVID-19, ICD-10: U07.1). MEWS, NEWS, and REMS were calculated for each patient, based as the main variables. Detailed parameters of MEWS, NEWS, and REMS are shown in Table 1.

### 2.4. Outcomes

The primary outcome of this study was the performance of MEWS, NEWS, and REMS to predict in-hospital mortality in COVID-19 patients.

### 2.5. Statistical Analyses

The independent t-test was used to analyze continuous data for between-group differences. Nominal variables were examined using Fisher’s exact test (expected value was <5 in one cell) or chi-square test as appropriate. Vital signs for each patient recorded during initial ED triage were used for calculating MEWS, NEWS, and REMS. Receiver operating characteristic (ROC) analysis was performed for all three EWS, and their discriminatory performance was estimated using the area under curve (AUC), which was calculated using the DeLong method [12,13]. The score with the largest Youden Index was determined as the optimal cut-off value for predicting COVID-19 in-hospital mortality for each EWS. Sensitivity, specificity, positive predictive value (PPV), and negative predictive value (NPV) for each EWS were calculated according to the optimal cut-off value.

The statistical software SPSS (version 26.0; SPSS Inc., Armonk, NY, USA) was used for all statistical analyses. A *p* value of <0.05 was defined as statistically significant.

### 2.6. Ethics Statement

The design and execution of this retrospective study was approved by the Institutional Review Board of MacKay Memorial Hospital (21MMHIS377e) on 13 December 2021. Informed consent was waived due to the retrospective nature of the study and the fact that the analysis used anonymous clinical data.

## 3. Results

**Figure 1:**
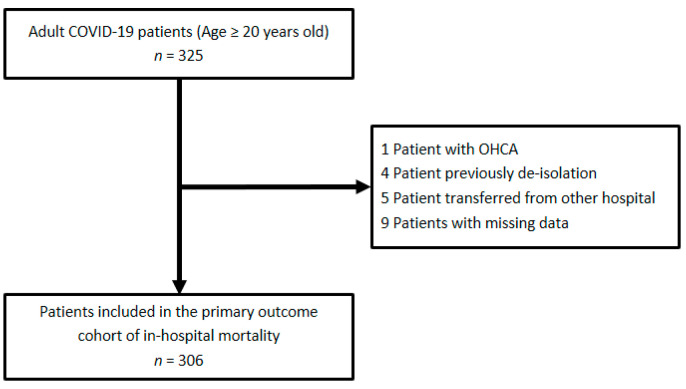
Flowchart of patients included in the study cohort. Abbreviation: OHCA: out-of-hospital cardiac arrest.

A total of 325 adult (age ≥ 20 years old) COVID-19 patients were hospitalized between May–July 2021 and were enrolled in this study. This study excluded patients with OHCA (*n* = 1), and those who had previously de-isolated from COVID-19 (*n* = 4), transferred from other hospital (*n* = 5), or were missing registry data (*n* = 9). Thus, data from 306 COVID-19 patients was included for analyses. A flowchart depicting patient inclusion and exclusion is provided in Figure 1.

Baseline characteristics of all 306 patients are presented in Table 2. Specifically, mean age of the cohort was 61.07 ± 15.12 years; 52.9% were male; and in-hospital mortality due to COVID-19 infection occurred in 35 (11.4%) patients after admission. Cases were categorized into mortality or survival groups, and a comparison of the two groups revealed that mean age was higher in the mortality group (69.7 vs. 59.9 years, *p* < 0.05), which also contained a larger proportion of males (65.7% vs. 51.2%, *p* < 0.05). Further, the mortality group also had a meaningfully higher prevalence of hypertension (60% vs. 31%, *p* < 0.05), diabetes mellitus (43% vs. 23%, *p* < 0.05), coronary artery disease (26% vs. 9%, *p* < 0.05), heart failure (11% vs. 2%, *p* < 0.05), and chronic kidney disease (40% vs. 5%, *p* < 0.05).

A comparison of vital signs at initial presentation revealed no difference in body temperature, diastolic blood pressure, or mean arterial pressure; however, the mortality group was associated with a lower Glasgow Coma Scale score (13.8 vs. 14.7, *p* < 0.05) and pulse oximetry (SpO2) level (88.6% vs. 95.4%, *p* < 0.05) but recorded higher heart rate (103.2 vs. 92 bpm, *p* < 0.05) and respiratory rate (21.9 vs. 19.6 bpm, *p* < 0.05). Notably, the mortality group had significantly higher REMS (8.46 vs. 4.87, *p* < 0.05), NEWS (6.06 vs. 3.25, *p* < 0.05), and MEWS (3.49 vs. 2.22, *p* < 0.05) values.

**Figure 2:**
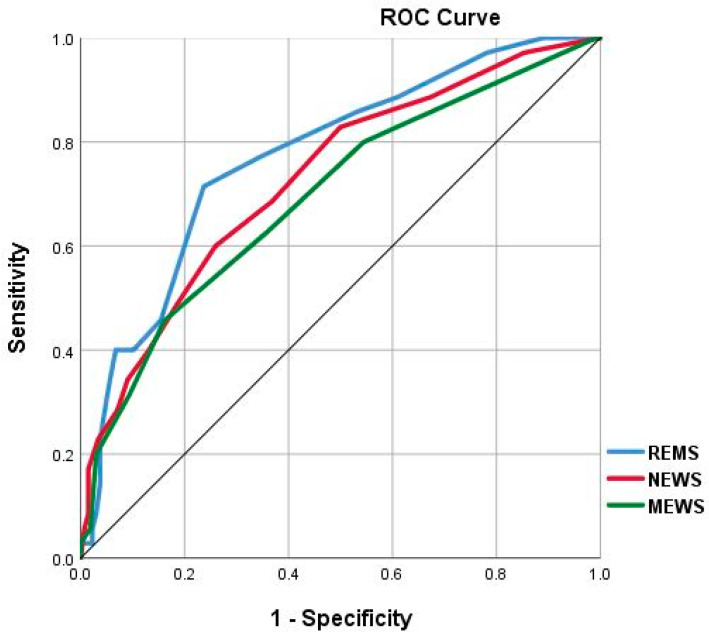
ROC curves for the early warning scores. Abbreviations: REMS: Rapid Emergency Medicine Score; NEWS: National Early Warning Score; MEWS: Modified Early Warning Score.

An analysis of the discriminant ability of REMS, NEWS, and MEWS for predicting in-hospital mortality among COVID-19 patients showed that REMS had the highest AUC (0.773, 95% CI: 0.69–0.85), followed by NEWS (0.730, 95% CI: 0.64–0.82) and MEWS (0.695, 95% CI: 0.60–0.79). The ROC of all three EWS are shown in Figure 2. Notably, all the three EWS evaluated achieved statistical significance compared to the null hypothesis, i.e., all three scores could adequately identify patients at risk of in-hospital mortality. The optimal cut-off value for REMS for predicting in-hospital mortality of COVID-19 patients was determined to be 6.5, which had a sensitivity of 71.4%, a specificity of 76.3%, PPV of 28.0%, and NPV of 95.4%. Data on cut-off values, sensitivity, specificity, PPV, and NPV for all three EWS are listed in Table 3.

## 4. Discussion

To the best of our knowledge, this is the first study to compare the performance of the three most utilized EWS for predicting in-hospital mortality among COVID-19 patients. The results described here show that REMS has the greatest predictive value among the three EWS tested, implying that REMS can help emergency physicians rapidly identify at-risk patients using only basic bedside vital signs.

This study used EWS to predict in-hospital mortality of COVID-19 patients rather than severity scores such as Acute Physiology and Chronic Health Evaluation II (APACHE II) because the latter require laboratory data over a 24-h period. We have also noticed that several COVID-19 severity scores were introduced in the pandemic, which required detailed past history, vital signs, and laboratory data [9]. In contrast, EWS permit immediate risk stratification and can be incorporated into a rapid response system to ensure timely intervention and avoid future adverse events [2]. Previous studies have demonstrated the importance of EWS in identifying COVID-19 patients at risk of deterioration [14,15,16,17]; however, a comparison of the three most utilized indices has not been reported.

MEWS was first described in 2001 and was prospectively validated in 709 patients who were hospitalized from the ED [3]. In that study, a MEWS value greater than 5 was found to be associated with increased intensive care unit admission and mortality [3], and another report has stated that the prognostic ability of MEWS is similar to that of APACHE II, the pneumonia severity index, and the sequential organ failure assessment score [18]. A retrospective study in 339 patients from Turkey calculated a MEWS composite AUC of 0.833 for 28-day mortality in COVID-19 patients [19]; in contrast, this study reports that MEWS only has fair discriminatory ability (AUC = 0.695). One reason for this discordance in results could be the fact that the previous study did not follow up patients throughout their hospitalization; rather, they used 28-day mortality as the endpoint [19]. Furthermore, in our study, MEWS was inferior to NEWS and REMS, probably because SpO2 is not included in MEWS, even though COVID-19 mainly affects the respiratory system and can cause excessive desaturation in severe cases [1].

NEWS was developed in 2012 as a standardized EWS for the National Health Service of the United Kingdom, was validated in a large population (n = 198,755), and boasts an AUC of 0.894 for patient death in the original study [4]. Subsequently, a new version, NEWS 2, was introduced in 2017, but recent studies have indicated that NEWS remains superior to NEWS 2 [2,20]. One study in French has revealed that NEWS is an accurate predictor of both ICU transfer and hospital mortality in COVID-19 patients [16], and that NEWS ≥6 at admission is an indicator of possible clinical decline during hospitalization [16]. Another study in Indonesia validated the performance of NEWS 2 in 112 patients with COVID-19. This study revealed a promising result that NEWS 2 can predict the death of patients with COVID-19. However, this cross-sectional study was confined to a smaller population [21]. Further investigation on discrimination comparing other EWS and NEWS 2 may be conducted on patients with COVID-19. In our cohort, NEWS showed an AUC of 0.730 for in-hospital mortality in COVID-19 patients, and we posit that NEWS demonstrated a higher AUC compared to MEWS because the former includes SpO2 as an additional physiological parameter. However, NEWS does not include age, and we have noticed that geriatric patients are more vulnerable to COVID-19 [9,22]. Thus, REMS, which includes age as a parameter, is better than both NEWS and MEWS [14,17].

REMS was first reported in 2004 in a prospective cohort study of 12,006 nonsurgical patients admitted from the ED and was demonstrably superior to another EWS—namely, the RAPS. [5] One study investigated the accuracy of REMS in predicting 30-day mortality in geriatric COVID-19 patients and reported that it is better than both RAPS and MEWS [17]. Similarly, other reports have also found REMS to be preferable to MEWS in COVID-19 patients aged younger than 65 years [14]. This study also reveals similar results, i.e., that among the three EWS evaluated, REMS is a better predictor compared to NEWS or MEWS, probably because it incorporates both age and SpO2 [3,4,5].

There were some limitations in this study. First, this was a retrospective study and there were instances of incomplete data (5.8%; 19/325). However, this value is less than 10%, which is acceptable. Second, this was a single-center study with a relatively small cohort of COVID-19 patients. Nonetheless, this is the first study to compare the three most used EWS. Additional multi-center prospective studies are needed to validate the predictive accuracy of these three EWS. Third, our study population had no complete vaccination in 2021. Only 69% of the population received the first dose of vaccination, and 63% achieved the second injection by January 2023 worldwide, although vaccination is widely available at present. Even now, our study could represent patients with COVID-19 with no complete vaccination. However, a national health database study is warranted to investigate patients with COVID-19 who are fully vaccinated.

## 5. Conclusions

This study evaluated the ability of REMS, NEWS, and MEWS to predict in-hospital mortality in COVID-19 patients admitted through the ED, and our results indicate that REMS is superior and that it may be used by emergency physicians for risk stratification of COVID-19 patients.

## Figures and Tables

**Table 1:** Comparison of vital signs used in MEWS, NEWS, and REMS.

	4	3	2	1	0	1	2	3	4	5	6
Modified Early Warning Score (MEWS)											
SBP (mmHg)		≤70	71–80	81–100	101–199		≥200				
HR (bpm)			<40	41–50	51–100	101–110	111–129	≥130			
RR (bpm)			<9		9–14	15–20	21–29	≥30			
BT (‘C)			<35		35–38.4		≥38.5				
AVPU					A	V	P	U			
National Early Warning Score (NEWS)											
RR (bpm)		≤8		9–11	12–20		21–24	≥25			
SpO2 (%)		≤91	92–93	94–95	≥96						
Oxygen supple					No		Yes				
BT (°C)		≤35		35.1–36	36.1–38	38.1–39	≥39.1				
SBP (mmHg)		≤90	91–100	101–110	111–219			≥220			
HR (bpm)		≤40		41–50	51–90	91–110	111–130	≥131			
AVPU					A			V, P, U			
Rapid Emergency Medicine Score (REMS)											
Age					<45		45–54	55–64		65–74	>74
MAP (mmHg)	≤ 49		50–69		70–109		110–129	130–159	>159		
HR (bpm)	≤ 39	40–54	55–69		70–109		110–139	140–179	>179		
RR (bpm)	≤ 5		6–9	10–11	12–24	25–34		35–49	>49		
SpO2 (%)	<75	75–85		86–89	>89						
GCS	<5	5–7	8–10	11–13	>13						

Abbreviations: A: alert; GCS: Glasgow Coma Scale; BT: body temperature; HR: heart rate; RR: respiratory rate; SBP: systolic blood pressure; MAP: mean arterial pressure; SpO2: pulse oximetry; REMS: Rapid Emergency Medicine Score; NEWS: National Early Warning Score; MEWS: Modified Early Warning Score; P: painfully responsive; U: unresponsive; V: verbally responsive.

**Table 2:** Demographic characteristics and initial vital signs at triage of COVID-19 patients in the study cohort.

	All Patients (n = 306)	Non-Survivors (n = 35)	Survivors (n = 271)	*p* Value
Basic characteristics				
Age	61.07 ± 15.12	69.74 ± 10.18	59.87 ± 15.23	<0.05
Sex	52.90%	65.71%	51.15%	<0.05
Medical history				
HTN	34%	60%	31%	<0.05
DM	26%	43%	23%	<0.05
CAD	10%	26%	8%	<0.05
HF	3%	11%	2%	<0.05
CKD	9%	40%	5%	<0.05
Vital signs (mean ± SD)				
GCS	14.57 ± 1.82	13.83 ± 2.99	14.65 ± 1.63	<0.05
BT	37.38 ± 1.77	37.70 ± 1.06	37.33 ± 1.86	0.25
HR	93.17 ± 20.65	103.23 ± 21.70	92.09 ± 19.98	<0.05
RR	20.16 ± 7.14	21.86 ± 5.17	19.59 ± 4.48	<0.05
SBP	129.99 ± 24.78	139.46 ± 30.64	128.79 ± 23.62	<0.05
DBP	75.25 ± 13.33	75.94 ± 15.17	75.30 ± 13.03	0.789
MAP	93.50 ± 15.32	93.13 ± 14.69	97.11 ± 18.98	0.148
SpO2	94.64 ± 7.99	88.60 ± 13.78	95.42 ± 6.40	<0.05
Emergency warning scores (mean ± SD)				
REMS	5.32 ± 3.67	8.46 ± 3.97	4.87 ± 3.32	<0.05
NEWS	3.56 ± 3.11	6.06 ± 3.79	3.25 ± 2.81	<0.05
MEWS	2.51 ± 1.91	3.49 ± 1.98	2.22 ± 1.47	<0.05

Abbreviations: HTN: hypertension; DM: diabetes mellitus; CAD: coronary artery disease; HF: heart failure; CKD: chronic kidney disease; SD: standard deviation; GCS: Glasgow Coma Scale; BT: body temperature; HR: heart rate; RR: respiratory rate; SBP: systolic blood pressure; DBP: diastolic blood pressure; MAP: mean arterial pressure; SpO2: pulse oximetry; REMS: Rapid Emergency Medicine Score; NEWS: National Early Warning Score; MEWS: Modified Early Warning Score.

**Table 3:** Discriminant ability of the REMS, NEWS, and MEWS for in-hospital mortality of COVID-19 patients.

Score	AUC	95% CI	*p* Value	Cutoff Value	Sensitivity	Specificity	PPV	NPV	PLR	NLR
REMS	0.773	0.692–0.854	<0.05	> 6.5	71.4%	76.3%	27.9%	95.4%	3.01	0.37
NEWS	0.730	0.639–0.820	<0.05	> 4.5	60.0%	74.1%	23.0%	93.5%	2.32	0.54
MEWS	0.695	0.597–0.792	<0.05	> 3.5	45.7%	83.8%	26.6%	92.3%	2.82	0.65

Abbreviations: AUC: area under the curve; CI: confidence interval; PPV: positive predictive value; NPV: negative predictive value; PLR: positive likelihood ratio; NLR: negative likelihood ratio; REMS: Rapid Emergency Medicine Score; NEWS: National Early Warning Score; MEWS: Modified Early Warning Score.

## Data Availability

The data are not publicly available due to restrictions regarding the Ethical Committee Institution.
